# The Duration and Determinants of Anti-SARS-CoV-2 Immunoglobulin G in Cancer Patients with SARS-CoV-2 Infection: A Longitudinal Study

**DOI:** 10.1007/s00284-022-02933-2

**Published:** 2022-06-29

**Authors:** Yao Jiang, Yingchao Zhao, Guiling Li

**Affiliations:** Cancer Center, Union Hospital, Tongji Medical College, Huazhong University of Science and Technology, 1277 Jiefang Road, Wuhan, 430022 Hubei China

## Abstract

Patients with cancer have an increased risk of severe acute respiratory syndrome coronavirus 2 (SARS-CoV-2) infection and a high case-fatality rate. The duration of anti-SARS-CoV-2 immunoglobulin G (IgG) antibodies in cancer patients following SARS-CoV-2 infection has not been reported previously. We conducted a longitudinal study at a cancer center in Wuhan, China to determine the duration of the humoral immune response following SARS-CoV-2 infection in cancer patients and to determine factors associated with a short duration (< 6 months) of anti-SARS-CoV-2 immunoglobulin G (IgG). Of 2139 cancer patients screened, 78 with confirmed SARS-CoV-2 infection were included in this study. SARS-CoV-2 IgG antibodies were present for < 6 months in 39.7% of these patients. In addition, patients who received chemotherapy were more likely to have a short duration of anti-SARS-CoV-2 IgG (odds ratio 5.31, 95% confidence interval 1.09–26.02, *P* < 0.05). Our study suggests that cancer patients, especially those who were receiving chemotherapy, have a shorter anti-SARS-CoV-2 IgG duration following infection and therefore, should be prioritized for vaccination.

## Introduction

Coronavirus disease 2019 (COVID-19), caused by severe acute respiratory syndrome coronavirus 2 (SARS-CoV-2), has spread rapidly throughout the world since the first case was detected in December, 2019. Cancer patients are a vulnerable population during the COVID-19 pandemic as they have an increased risk of SARS-CoV-2 infection and high case-fatality rates owing to their immunosuppressed status [1–3]. However, knowledge of their immune response to infection is limited because published studies on the immunoglobulin G (IgG) response to SARS-CoV-2 infection have not included cancer patients [4, 5]. We reviewed medical records and clinical data of cancer patients with SARS-CoV-2 infection who were treated at the Cancer Center, Union Hospital, Wuhan, China. This study aimed to determine the duration of anti-SARS-CoV-2 IgG in cancer patients following SARS-CoV-2 infection and to identify risk factors associated with the duration of anti-SARS-CoV-2 IgG.

## Materials and Methods

### Study Design and Participants

This longitudinal cohort study was performed in the Cancer Center, Union Hospital, affiliated with the Tongji Medical College of Huazhong University of Science and Technology. Cancer patients with laboratory-confirmed SARS-CoV-2 infection were included from March 23 to April 30, 2020. The cut-off date of our study was June 2, 2020.

This study was approved by the Union Hospital of Huazhong University of Science and Technology ethics committee (20200258). The ethics committee granted a waiver of the requirement for informed consent because of the urgency of COVID-19.

### Data Collection and Materials

We retrieved clinical data including demographic features, clinical manifestations, cancer histories, stages, and treatments from medical records and telephone interviews. All data were reviewed independently by two physicians (YJ and YZ). Based on the TNM staging system, cancer stage was categorized as early or locally advanced (stage I–III) and metastatic or relapsed diseases (stage IV). Cancer treatments including surgery, chemotherapy, radiotherapy, immunotherapy, and targeted therapy were recorded.

Blood samples were collected and tested for anti-SARS-CoV-2 IgG using a commercially available colloidal gold qualitative immunochromatographic assay kit (Nanjing Vazyme Medical Technology Co, Nanjing, China). Mammalian cell-expressed recombinant antigens containing the N protein and the spike protein of SARS-CoV-2 were used to detect the anti-SARS-CoV-2 IgG and immunoglobulin M (IgM) antibodies. The test has a reported sensitivity and specificity of 86.6% and 96.8% for IgM, and 87.1% and 99.2% for IgG, respectively.

### Definitions

SARS-CoV-2 infection was diagnosed according to the criteria published by WHO. Symptomatic cancer patients with COVID-19 were diagnosed based on a positive reverse transcription-polymerase chain reaction (RT-PCR) test for SARS-CoV-2 RNA with clinical symptoms, including fever, cough, fatigue, dyspnea, muscle soreness, and diarrhea. Asymptomatic SARS-CoV-2 infection was diagnosed based on positive anti-SARS-CoV-2 IgG antibody tests without any COVID-19 symptoms.

### Statistical Analyses

For the descriptive analysis, continuous variables were presented as medians and interquartile ranges (IQRs), and categorical variables were presented as percentages. The *χ*^2^ test was used to test for the significance of differences between groups. Multivariable logistic regression analysis was performed to identify factors associated with a < 6 months duration of anti-SARS-CoV-2 IgG antibody. All statistical analyses were performed using SPSS Statistics Version 26.0 (IBM Corp., Armonk, NY, USA). Two-sided *P*-values < 0.05 were considered to be statistically significant.

## Results

### Patient Demographic and Clinical Characteristics

Of 2139 cancer patients admitted to the Cancer Center from March 23 to April 30, 2020, 89 (4.2%) were confirmed with symptomatic or asymptomatic SARS-CoV-2 infection. After excluding 11 patients without follow-up, 78 cancer patients were enrolled in this study and followed up until the cut-off date (June 2, 2020). Of these patients, 17 (21.8%) patients were symptomatic and 61 (78.2%) patients were asymptomatic. All 78 cancer patients had at least two serial measurements of anti-SARS-CoV-2 IgG and all had a positive first test. The median age was 58 years (interquartile range 51–64 years), and 34 (43.6%) patients were female. Comorbidities including hypertension, diabetes, and coronary heart disease were present in 22 patients (28.2%). The commonest malignancies were lung, colorectal, and cervical cancer; 42 patients (53.8%) had stage IV disease. Of the 78 cancer patients, 61 (78.2%) underwent antitumor therapy including chemotherapy (*n* = 37, 47.4%), immunotherapy (*n* = 8, 10.3%), radiotherapy (*n* = 6, 7.7%), surgery (*n* = 5, 6.4%), and targeted therapy (*n* = 5, 6.4%) between January 1, 2020 and the final anti-SARS-CoV-2 antibody test. As all patients were diagnosed between January 23 and March 10, 2020, the period between SARS-CoV-2 infection and the last anti-SARS-CoV-2 antibody test was less than 6 months (the cut-off date was June 2, 2020). No significant differences were observed in the duration of IgG antibodies between the groups in terms of age, symptoms, and comorbidities.

### Risk Factors for Early Antibody Disappearance

Overall, 31 (39.7%) patients reverted to IgG negative during the follow-up period. In the multivariable logistic regression analysis that included age, sex, symptomatic infection, cancer type, stage, treatment, and comorbidities, chemotherapy was the only factor identified as independently associated with the duration of anti-SARS-CoV-2 IgG antibody response (Table 1).

**Table 1:** Results of multivariable logistic regression analysis of factors associated with a short duration (< 6 months) of anti-SARS-CoV-2 IgG

	*n* (%)	OR	95% CI	*P*-value
Age, years				
≤ 60	47 (60.3%)	1.00 (ref)	–	–
> 60	31 (39.7%)	1.12	0.32–3.95	0.86
Gender				
Female	34 (43.6%)	1.00 (ref)	–	–
Male	44 (56.4%)	2.98	0.95–9.31	0.06
With or without symptoms				
Asymptomatic infection	61 (78.2%)	1.00 (ref)	–	–
Symptomatic infection	17 (21.8%)	0.73	0.19–2.87	0.65
Cancer type				
Lung cancer	33 (42.3%)	1.00 (ref)	–	–
Other types of cancer	45 (57.7%)	2.35	0.71–7.79	0.16
Stage				
I–III	36 (46.2%)	1.00 (ref)	–	–
IV	42 (53.8%)	2.07	0.64–6.72	0.23
Antitumor treatments				
None	17 (21.8%)	1.00 (ref)	–	–
Chemotherapy	37 (47.4%)	5.31	1.09–26.02	0.04*
Immunotherapy	8 (10.3%)	4.28	0.49–37.57	0.19
Radiotherapy	6 (7.7%)	2.21	0.23–21.48	0.50
Surgery	5 (6.4%)	5.85	0.44–78.33	0.18
Targeted therapy	5 (6.4%)	< 0.01	–	> 0.99
Comorbidities				
Without comorbidities	56 (71.8%)	1.00 (ref)	–	–
With comorbidities	22 (28.2%)	0.80	0.23–2.79	0.72

SARS-CoV-2 IgG, severe acute respiratory syndrome coronavirus 2 immunoglobulin G; OR, odds ratio; CI, confidence interval; ref, reference

*Means statistically significant difference

## Discussion

We determined the clinical characteristics and immune responses of 78 cancer patients with SARS-CoV-2 infection. Currently, there are limited data on the duration of SARS-CoV-2 IgG in cancer patients following SARS-CoV-2 infection. In this study, we found that IgG antibodies were present for < 6 months among 39.7% of the cancer patients with confirmed SARS-CoV-2 infection. Studies have reported that most patients who recovered from severe acute respiratory syndrome coronavirus (SARS-CoV) infection developed long-lasting immunity [6–8]. A model of SARS-CoV-2 transmission projected that duration of immunity to SARS-CoV-2 is likely to be approximately 40 weeks [9]. Dan reported that both SARS-CoV-2 Spike IgG and SARS-CoV-2 neutralizing antibodies were persistent in 90% of convalescents at 6–8-month post-symptom onset [10]. One longitudinal study of antibody persistence reported that anti-spike IgG remained positive at 180 days in 94% of UK health-care workers [11]. Another seroprevalence study showed that SARS-CoV-2 IgG was persistent in 84% of patients for nearly 6 months after COVID-19 infection [12]. Our study suggests that cancer patients may have a shorter anti-SARS-CoV-2 IgG duration than non-cancer individuals after SARS-CoV-2 infection. A possible explanation could be that a majority of cancer patients were immunocompromised because of cancer or cancer treatments. Studies have suggested that IgG antibody levels fell faster in younger adults and asymptomatic patients [13]. In our study, no significant differences were observed in the duration of IgG antibodies between the groups in terms of age, symptoms, and comorbidities. Furthermore, our study demonstrated that cancer patients who underwent chemotherapy were more likely to have short-term immunity to SARS-CoV-2. This could be due to chemotherapy-induced immunosuppression. Esperança-Martins et al. reported that among 19 cancer patients infected with SARS-CoV-2, chemotherapy within 14 days before RT-PCR positivity for SARS-CoV-2 was associated with weak serological responses. Notably, the majority of cancer patients included in this study were symptomatic (94.7%) [14].

However, our study has several limitations. First, the study was conducted in a single cancer center with a small sample size. Therefore, the results should be interpreted with caution. Second, since the study was retrospective, several factors including the frequency and variable time points of antibody testing confounded the accurate estimation of antibody response duration in this study.

## Conclusion

In conclusion, during the COVID-19 pandemic, cancer patients, especially those who were receiving chemotherapy, have a shorter anti-SARS-CoV-2 IgG duration following infection and therefore, should be prioritized for vaccination. Further studies are needed to investigate the long-term duration of anti-SARS-CoV-2 IgG in cancer patients.

## Data Availability

The data used to support the findings of this study are available from the corresponding author on reasonable request.
